# Genome editing in clinical practice: A model study for next-gen hematopoietic cell transplants in hematologic malignancies

**DOI:** 10.1016/j.omtm.2024.101210

**Published:** 2024-02-26

**Authors:** Patrick Derigs, Carsten Müller-Tidow

**Affiliations:** 1 Department of Internal Medicine V, University Hospital Heidelberg, Heidelberg, Germany; 2 DNA Vector Lab, German Cancer Research Center (DKFZ), Heidelberg, Germany; 3 German Cancer Consortium (DKTK) and German Cancer Research Center (DKFZ)/National Center for Tumor Diseases (NCT), Heidelberg, Germany

## Main text

Immunotherapies are precise antigen-specific approaches that revolutionize cancer treatment. The goal is to specifically target cancer cells while minimizing effects on healthy cells. Such therapy approaches include monoclonal antibodies with and without drug payloads, bispecific T cell engagers, and chimeric antigen receptor (CAR) T cells. However, many antigens, like the anti-acute myeloid leukemia (AML) antigen CD33, are not only expressed on hematologic malignancies but also on normal hematopoietic cells. Recently, approaches utilizing gene-editing tools like classical CRISPR-Cas9 or base editing have been presented to render target antigens on healthy hematopoietic stem and progenitor cells (HSPCs) incapable of antibody or CAR binding, resulting in minimal off-tumor toxicity after transplantation of these gene-edited HSPCs.^1^^,^^2^^,^^3^^,^^4^

First-in-human clinical evaluation of a next-generation hematopoietic cell transplant (HCT) in patients with AML is currently ongoing (ClinicalTrials.gov: NCT04849910). In the recent study of Lydeard et al. published in *Molecular Therapy Methods and Clinical Development*, data on the preclinical development program and the scale-up process leading to this clinical trial are presented.^5^ The authors used classical CRISPR-Cas9 to genetically ablate CD33 from healthy donor HSPCs by means of a complete gene knockout, creating an HSPC transplant product that is resistant to anti-CD33 drug cytotoxicity. They describe a comprehensive program including studies of *in vitro* differentiation and functional assays, assessment of off-target editing events, and pharmacology studies using mouse xenograft models. Notably, the presented results led to the investigational new drug (IND) approval of the FDA, displaying a blueprint for other groups preparing clinical trials with gene-edited HCTs in hematologic malignancies.

In March 2022, the FDA published a draft guidance with recommendations regarding information that should be provided in an IND application of human gene therapy products incorporating genome editing (GE) of human somatic cells.^6^ Lydeard et al. have demonstrated data that support their product in a science-based approach weighing the benefits and risks. Unintended consequences of on- and off-target editing have been addressed systematically, forming a model study that guides other sponsors toward successful IND applications.

**Figure 1:**
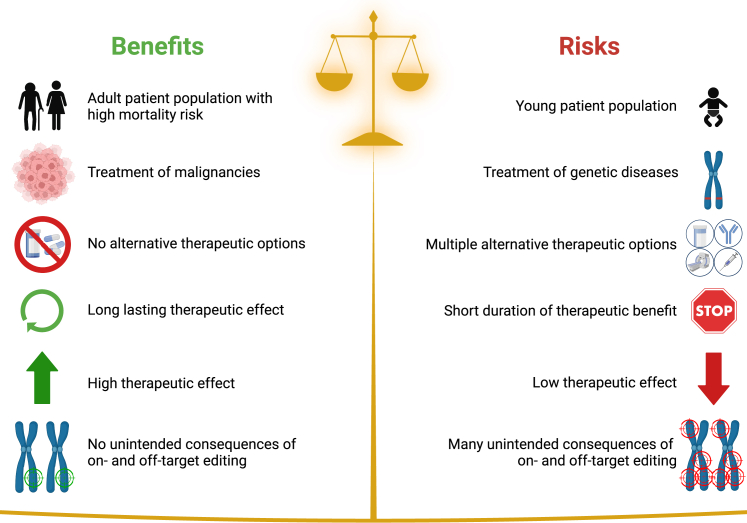
Science-based approach weighing benefits and risks of treatments incorporating genome editing

Especially in relapsed or refractory hematologic malignancies like AML, there is an urgent medical need for new therapeutic approaches. Promising new cancer therapies incorporating GE are coming up at a fast pace. For regulatory authorities, it is important to focus on the benefit-risk profile of each GE product (Figure 1). This assessment should consider indication and disease situation (earlier vs. later treatment lines), patient population (older vs. younger patients), and anticipated therapeutic effect (correction of genetic diseases vs. enabling targeted therapy of malignancies).

Next-generation cell transplantation products incorporating GE can be used in a range of therapeutic approaches treating malignant and non-malignant diseases. With their study, Lydeard et al. provide a road map for new GE projects, accelerating development of new INDs and helping sponsors to calculate their preclinical programs. Some level of planning security is important for these projects since they are ambitious, time consuming, and expensive, with uncharted territory. Concerning the scale of the program of Lydeard et al., academic groups with less financial resources will not be able to conduct similar studies. They were able to produce 30 batches of clinical-scale GE products, underlining their potency of industrial manufacturing.

GE of HSPCs is a very promising avenue for next-generation cellular therapies. Transplantation of autologous and allogeneic stem cells is a very well-established cell therapy, which yearly benefits many thousands of patients worldwide. The procedure inherent risks are well defined, with less than 1% mortality in autologous HCTs. These established standard procedures of hematopoietic stem cell transplantation can be used in clinical practice. Of course, GE might pose additional risks beyond the transplantation procedure. Therefore, Lydeard et al.’s approach makes the most sense for patients at high risk of dying of malignant disease. Upon successful establishment of such therapies in patients with cancer, the next step could be to cure other acquired or even inherited diseases. Accordingly, the recent approval of the first CRISPR-based therapy for sickle cell disease represents an important milestone in next-generation medicine.^7^ Further, base editing provides additional opportunities for rewriting the genetic code. It may allow the simultaneous correction or enhancement of multiple genetic loci. Thus, we envision a broad range of applications for clinical medicine. Today, the future of genomic medicine appears bright. The seminal study by Lydeard et al. should help the field on the way toward clinical GE in hematopoietic stem cells.

## References

[1] Kim M.Y., Yu K.R., Kenderian S.S., Ruella M., Chen S., Shin T.H., Aljanahi A.A., Schreeder D., Klichinsky M., Shestova O. (2018). Genetic Inactivation of CD33 in Hematopoietic Stem Cells to Enable CAR T Cell Immunotherapy for Acute Myeloid Leukemia. Cell.

[2] Borot F., Wang H., Ma Y., Jafarov T., Raza A., Ali A.M., Mukherjee S. (2019). Gene-edited stem cells enable CD33-directed immune therapy for myeloid malignancies. Proc. Natl. Acad. Sci. USA.

[3] Casirati G., Cosentino A., Mucci A., Salah Mahmoud M., Ugarte Zabala I., Zeng J., Ficarro S.B., Klatt D., Brendel C., Rambaldi A. (2023). Epitope editing enables targeted immunotherapy of acute myeloid leukaemia. Nature.

[4] Wellhausen N., O'Connell R.P., Lesch S., Engel N.W., Rennels A.K., Gonzales D., Herbst F., Young R.M., Garcia K.C., Weiner D. (2023). Epitope base editing CD45 in hematopoietic cells enables universal blood cancer immune therapy. Sci. Transl. Med..

[5] Lydeard J.R., Lin M.I., Ge H.G., Halfond A., Wang S., Jones M.B., Etchin J., Angelini G., Xavier-Ferrucio J., Lisle J. (2023). Development of a gene edited next-generation hematopoietic cell transplant to enable acute myeloid leukemia treatment by solving off-tumor toxicity. Mol. Ther. Methods Clin. Dev..

[6] U.S. Department of Health and Human Services, Food and Drug Administration, Center for Biologics Evaluation and Research, Human Gene Therapy Products Incorporating Human Genome Editing - Draft Guidance for Industry, in *Docket Number FDA-2021-D-0398*. March 2022.

[7] ASH. Available from: https://www.hematology.org/newsroom/press-releases/2023/ash-statement-on-fda-approval-of-new-sickle-cell-disease-gene-therapies.

